# The Diagnostic and Differential Diagnosis Utility of Cerebrospinal Fluid ***α***-Synuclein Levels in Parkinson's Disease: A Meta-Analysis

**DOI:** 10.1155/2015/567386

**Published:** 2015-07-29

**Authors:** Bo Zhou, Min Wen, Wen-Feng Yu, Chun-Lin Zhang, Ling Jiao

**Affiliations:** ^1^Department of Biology, Guiyang Medical University, Guizhou 550004, China; ^2^Department of Anatomy, Guiyang Medical University, Guizhou 550004, China; ^3^Department of Molecular Biology, Guiyang Medical University, Guizhou 550004, China; ^4^Department of Neurology, Affiliated Hospital, Guiyang Medical University, Guizhou 550004, China

## Abstract

Several recent studies showed that *α*-syn might be a potential diagnostic biomarker for PD in human cerebrospinal fluid (CSF), but the results were inconsistent. The purpose of this meta-analysis was to investigate the diagnostic and differential diagnosis efficacy of CSF *α*-syn in PD. Studies which measured CSF *α*-syn or *α*-syn oligomers in patients with PD and met the inclusion criteria were included in the analysis. Results of the meta-analysis indicated that mean concentration of CSF *α*-syn was significantly lower in PD compared to controls and significantly higher in PD compared to multiple system atrophy (MSA). No significant difference in mean concentration of CSF *α*-syn was found between PD and dementia with Lewy bodies (DLB). Mean concentration of CSF *α*-syn was slightly decreased in PD compared to progressive supranuclear palsy (PSP). Mean concentration of CSF *α*-syn oligomers was significantly higher in PD than control. These results support the findings that CSF *α*-syn may be a potential diagnostic and differential diagnosis biomarker in PD compared to control and MSA but not DLB. Furthermore, *α*-syn oligomer may represent a better biomarker for diagnosis of PD.

## 1. Introduction

Parkinson's disease (PD) is the second most common neurodegenerative disease after Alzheimer's disease, affecting 1 and 5% of the population at 65 and 85 years of age, respectively [1]. Loss of dopaminergic neurons in the substantia nigra pars compacta and accumulation of intracellular inclusions (Lewy bodies) constitute basic pathological features of PD [2]. Currently, diagnosis of PD primarily depends on clinical criteria (the duration of disease, the clinical features, and the experience of the diagnosing physician) [3]. This approach, however, presents some limitations. The clinical criteria for diagnosis of PD encompass full-blown disease, when most dopaminergic neurons have undergone degeneration [4]. Moreover, differential diagnosis from other synucleinopathies (such as MSA and DLB) and tauopathies with atypical Parkinsonism (such as PSP and corticobasal degeneration (CBD)) can be rather challenging due to overlapping symptoms, particularly during the early disease stages. Early diagnosis of PD is a priority given current efforts towards development of disease-modifying therapies for this disorder [5, 6]. To the best of our knowledge, there is no established laboratory test or biomarker that can reliably and specifically identify PD as of today. So, it is important to search for an accurate and reliable marker for diagnosing PD early and differential diagnosis as well.

*α*-synuclein (*α*-syn) is an attractive candidate for a potential marker of PD because it is strongly linked with the pathogenesis of both familial and sporadic forms of this disease. *α*-syn is prominently expressed in the central nervous system and has been identified as the main component of the Lewy bodies [7, 8]. Recently, in vivo and in vitro studies suggest that soluble *α*-syn oligomers rather than the monomeric or fibrillar protein are the main pathological form of *α*-syn in the PD brain [9, 10]. CSF *α*-syn is principally derived from neurons in the central nervous system [11]. So we surmised that the main form of CSF *α*-syn in PD was oligomers and their levels were significantly higher in PD compared to control. This assumption was confirmed by Park's research [12, 13]. Several studies have explored the potential use of *α*-syn as a PD diagnostic biomarker in human CSF, but the results are inconsistent: some studies have shown a clear trend of lower total CSF *α*-syn in PD and other types of Parkinsonism such as MSA, DLB, and PSP, whereas others show no significant variation. To evaluate the potential diagnostic and differential diagnosis effect of CSF *α*-syn and *α*-syn oligomers in PD, a meta-analysis was conducted in this present study by combining all available data together.

## 2. Methods

### 2.1. Data Sources

A computerized search of Pubmed, Web of Science (v5.15), the Cochrane Library, and China National Knowledge Infrastructure (CNKI) from January 1980 to Dec 2014 was conducted using the following search strategy: (1) (“parkinson's disease” or “PD”), (“cerebrospinal fluid” or “CSF”), and (“*α*-synuclein” or “*α*-syn” or “alpha-synuclein” or “alpha-syn”); (2) (“parkinson's disease” or “PD”), (“cerebrospinal fluid” or “CSF”), and (“*α*-synuclein oligomers” or “*α*-syn oligomers” or “alpha-synuclein oligomers” or “alpha *α*-syn oligomers”), respectively. The language was restricted to English or Chinese. The reference lists of relevant studies were also searched for possible studies meeting criteria. Where there was an initial disagreement, discussion among researchers established universal agreement on studies to be included. A flowchart of information pertaining to identification, screening, eligibility, and final studies included was constructed according to PRISMA guidelines.

### 2.2. Inclusion and Exclusion Criteria

Studies comparing CSF *α*-syn or *α*-syn oligomers in patients with PD, controls (healthy control and nonneurodegenerative disease controls), or other Parkinsonism subjects were included. The inclusion criteria were (1) case-control studies design; (2) all patients of PD meeting the UK Brain Bank diagnostic criteria (the item not included in analysis of *α*-syn oligomers); and (3) data of the CSF *α*-syn or *α*-syn oligomers levels being available in the report or obtainable from the corresponding author. The exclusion criteria were studies which only included (1) CSF erythrocyte count >500/mm^3^ or hemoglobin >200 ng/mL; (2) duplicate of earlier publications; (3) papers focused on familial PD; and (4) the CSF *α*-syn from in vitro, animal model, and autopsy samples.

### 2.3. Data Extraction

Duplicates were deleted and the title and abstract of each article were scanned for relevance independently by two researchers. The full texts of potentially relevant studies were then retrieved and assessed for eligibility by established criteria detailed above.

Data for each individual CSF *α*-syn or *α*-syn oligomers assessed in eligible studies were extracted into an Excel spreadsheet (author, year of publication, sample size, mean or median, standard deviation or interquartile range, and method). One author (Bo Zhou) extracted all data. The method used to extract data was independently verified by another author (Min Wen). Data were then rechecked for accuracy (Bo Zhou).

### 2.4. Statistical Analysis

All statistical analyses were performed using standardized mean difference (SMD) methodology in Review Manager 5.3.3 and STATA 11.0. The medians and interquartile range in some studies were converted to means and standard deviation in accordance with the protocol provided by Hozo et al. [14]. We used 95% confidence interval (CI) to gauge the precision of the summary estimates. Overall heterogeneity was assessed using Cochran's *Q* statistic (*P* value was greater than 0.10 on the *Q* test, which reflects a lack of heterogeneity among studies) and *I* ^2^ (values of more than 50% as “considerable heterogeneity”). A random-effects model or fixed-effects model was used to calculate pooled SMD in the presence or absence of heterogeneity, respectively. We performed sensitivity analyses to assess the influence of individual studies on the pooled estimate. Publication bias was investigated using funnel plots, with a roughly symmetrical distribution on either side of the summary estimate, suggesting a lack of bias.

## 3. Results

### 3.1. Characteristics of *α*-Syn Studies Included in Meta-Analysis

**Figure 1:**
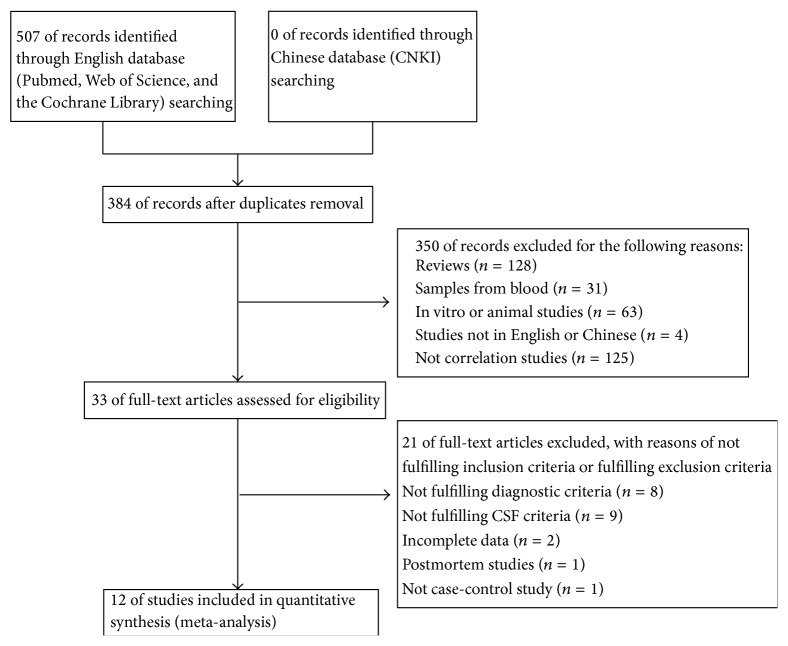
Flow diagram of the study selection process used for the meta-analysis of *α*-syn.

All studies were identified and the number of studies which were subsequently included or excluded is illustrated as a flow diagram in Figure 1. A total of 507 studies were identified from the databases searched and 123 duplicate studies were removed, while 384 individual studies remained. A further 351 papers were excluded after screening by title and abstract. Full-text review of the remaining 33 studies rejected those studies which did not meet the inclusion criteria or met the exclusion criteria (Chi-square test shows that the extracted information by two authors was consistent, *P* > 0.05). A total of 12 studies, including 1131 patients, met stringent search criteria and were included in the final review. A summary of data extracted from these studies is compiled in Table 1.

### 3.2. Characteristics of *α*-Syn Oligomers Studies Included in Meta-Analysis

**Figure 2:**
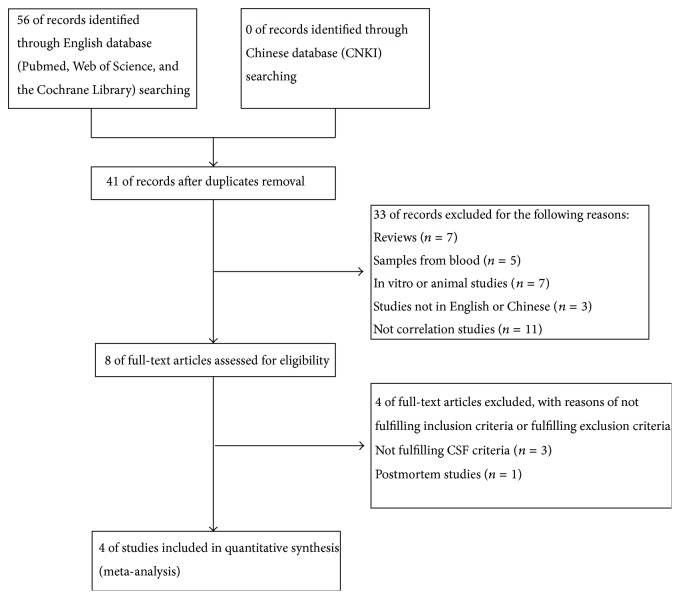
Flow diagram of the study selection process in the meta-analysis of *α*-syn oligomers.

Figure 2 shows a flow diagram of all studies identified and the number of studies which were subsequently included or excluded. A total of 56 studies were identified from the databases searched, 15 duplicate studies were removed, and 41 individual studies remained. A further 33 papers were excluded after screening by title and abstract. Full-text review of the remaining three studies rejected those which did not meet the inclusion criteria or met the exclusion criteria (Chi-square test shows that the extracted information by two authors was consistent, *P* > 0.05). A total of four studies, including 168 patients, met stringent search criteria and were included in the final review. A summary of data extracted from these studies is compiled in Table 2.

### 3.3. Concentration of CSF *α*-Syn in PD versus Control

**Figure 3:**
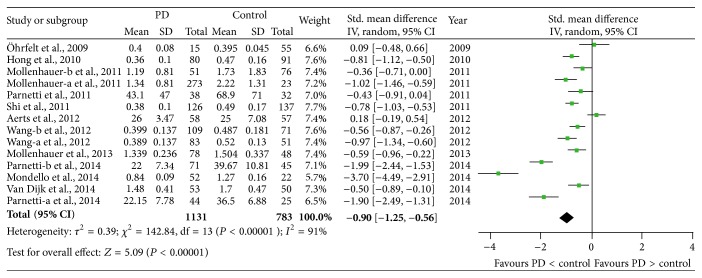
Forest plot of CSF *α*-syn levels in PD compared to controls.

Twelve studies (including 1131 patients and 783 controls) were used in this analysis. These studies had highly significant heterogeneity (*P* < 0.00001, *I* ^2^ = 91%), so a random-effect model was used to calculate pooled SMD. Forest plot (Figure 3) showed that the diamond was on the left side of the vertical line and did not intersect with the line, which demonstrates a significantly lower mean concentration of CSF *α*-syn in patients with PD compared to controls (SMD: −0.90, 95% CI: [−1.25, −0.56], *Z* = 5.09, *P* < 0.00001). To explore the source of the heterogeneity, a metaregression analysis (mixed-effect model) was conducted; the results showed that the age of patients, the type of control (healthy control or nonneurodegenerative disease controls), and the detection methods had lower effect on the heterogeneity (*P* > 0.05). Thus, the heterogeneity might be associated with the disease duration and severity (the original data are incomplete and led to no further analysis). At the same time, we compared the Coefficient of Variation (CV%) in each assay; the results showed that most assays were more concentrated; the CV% was in the range of 11–37%, but Mollenhauer et al.'s [24] and Parnetti et al.'s [23] studies were discrete (59–109%). We also compared the CV% in each detection method; the CV% in ELISA (11–109%) was more discrete than Luminex (25–37%).

### 3.4. Concentration of CSF *α*-Syn in PD versus MSA

**Figure 4:**
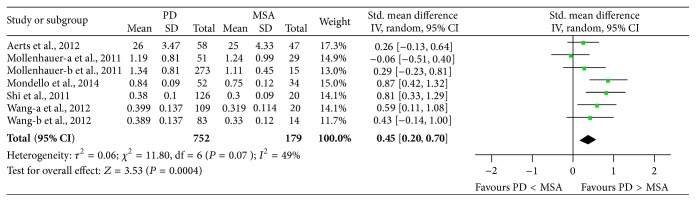
Forest plot of CSF *α*-syn levels in PD compared to MSA.

Five studies (included 752 PD and 179 MSA patients) were used in this analysis. These studies had moderate heterogeneity (*P* = 0.07, *I* ^2^ = 49%), so we used a random-effect model to calculate pooled SMD. Forest plot (Figure 4) showed that the diamond was on the right side of the vertical line and did not intersect with the line, which demonstrates a significantly higher mean concentration of CSF *α*-syn in patients with PD compared to MSA (SMD: 0.45, 95% CI: [0.20, 0.70], *Z* = 3.53, *P* = 0.0004).

### 3.5. Concentration of CSF *α*-Syn in PD versus DLB

**Figure 5:**
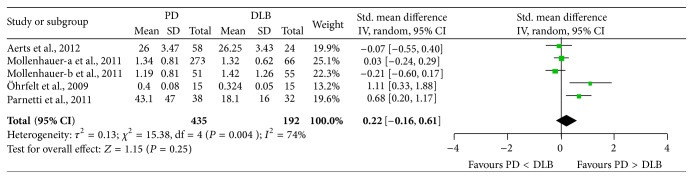
Forest plot of CSF *α*-syn levels in PD compared to DLB.

Four studies (including 435 PD and 192 DLB patients) were used in this analysis. These studies were not homogeneous (*P* = 0.004, *I* ^2^ = 74%), so we used a random-effect model to calculate pooled SMD. Forest plot (Figure 5) showed that the diamond was intersecting with the vertical line, which demonstrates no significant difference in CSF *α*-syn concentration between PD and DLB (SMD: 0.22, 95% CI: [−0.16,0.61], *Z* = 1.15, *P* = 0.25).

### 3.6. Concentration of CSF *α*-Syn in PD versus PSP

**Figure 6:**
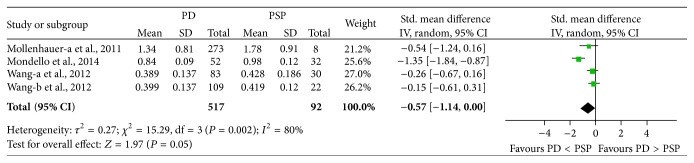
Forest plot of CSF *α*-syn levels in PD compared to PSP.

Three studies (including 517 PD and 92 PSP) were used in this analysis. These studies were not homogeneous (*P* = 0.001, *I* ^2^ = 80%), so we used a random-effect model to calculate pooled SMD. Forest plot (Figure 6) showed that the diamond was on the left side of vertical line and infinitely close to it, which demonstrates that the mean concentration of CSF *α*-syn was slightly decreased in PD compared to PSP, and the difference was marginally significant (SMD: −0.57, 95% CI: [−1.14, −0.00], *Z* = 1.97, *P* = 0.05).

### 3.7. Concentration of CSF *α*-Syn Oligomers in PD versus Control

**Figure 7:**
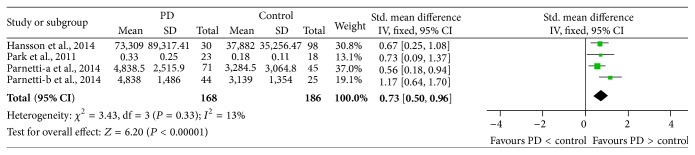
Forest plot of CSF *α*-syn oligomers levels in PD compared with control.

Four studies (included 168 PD and 186 controls) were used in this analysis. These studies were homogeneous (*P* = 0.33, *I* ^2^ = 13%), so we used a fixed-effect model to calculate pooled SMD. Forest plot (Figure 7) showed that the diamond was on the right side of the vertical line and did not intersect with the line, which demonstrated a significantly higher mean concentration of CSF *α*-syn oligomers in patients with PD compared to controls (SMD: 0.73, 95% CI: [0.50, 0.96], *Z* = 6.20, *P* < 0.00001).

### 3.8. Sensitivity Analysis

**Figure 8:**
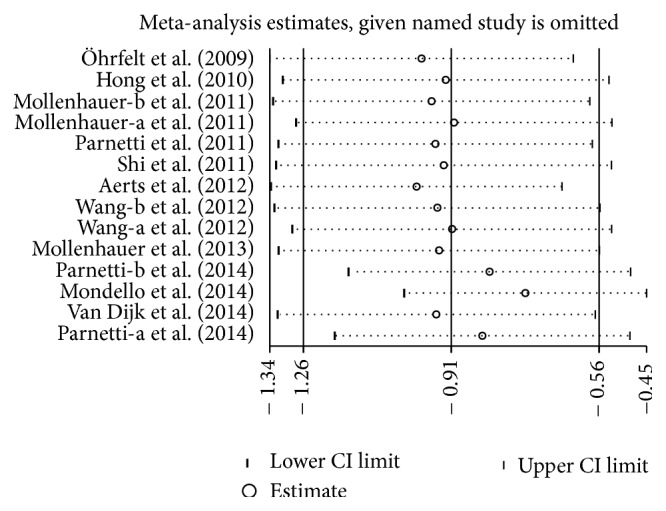
Sensitivity analysis of CSF *α*-syn levels in PD compared to control.

**Figure 9 fig9:**
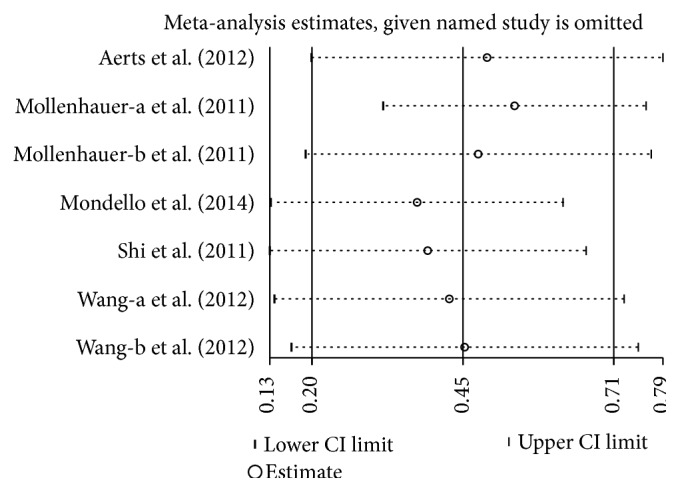
Sensitivity analysis of CSF *α*-syn levels in PD compared to MSA.

**Figure 10 fig10:**
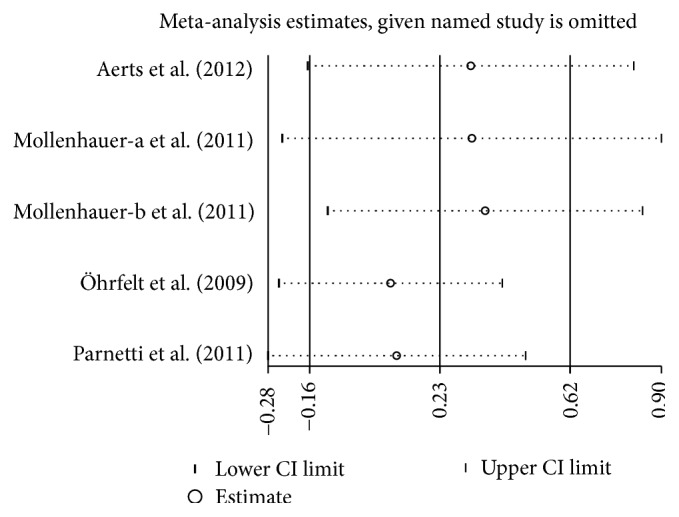
Sensitivity analysis of CSF *α*-syn levels in PD compared to DLB.

**Figure 11 fig11:**
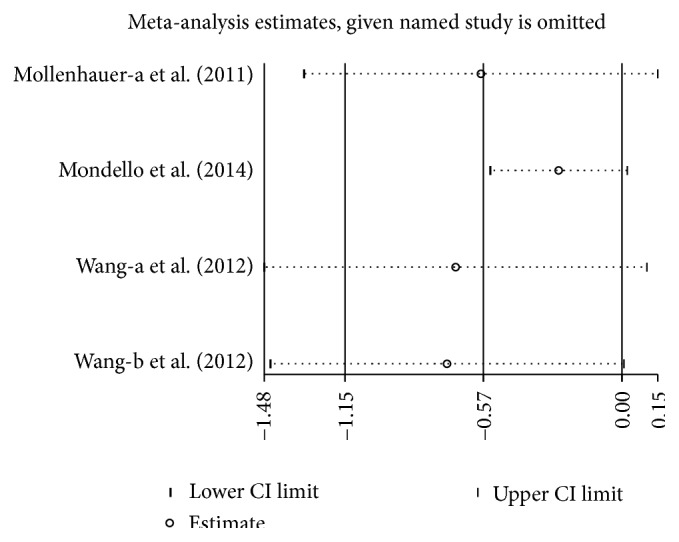
Sensitivity analysis of CSF *α*-syn levels in PD compared to PSP.

**Figure 12:**
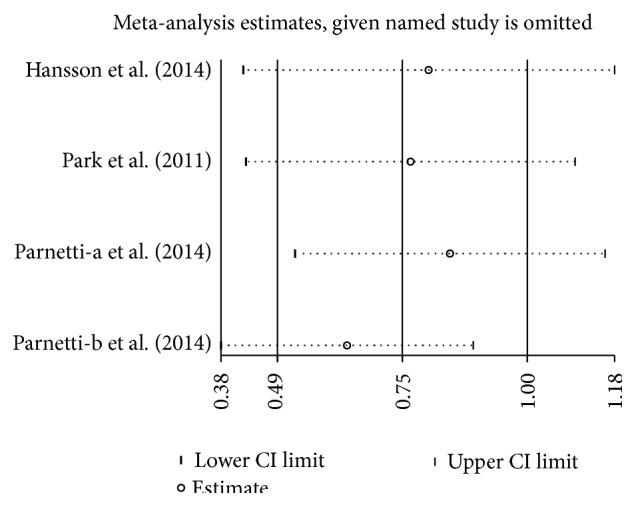
Sensitivity analysis of CSF *α*-syn oligomers levels in PD compared to control.

A sensitivity analysis was carried out for each meta-analysis to assess the influence of every single study. There was no significant change in the pooled SMD or 95% CI when removing one study at the time from all meta-analysis except in analysis between PD and PSP (Figures 8 [Fig fig9] [Fig fig10] [Fig fig11]–12), which indicates that the result of analysis between PD and PSP was not stable and reliable.

### 3.9. Publication Bias

**Figure 13:**
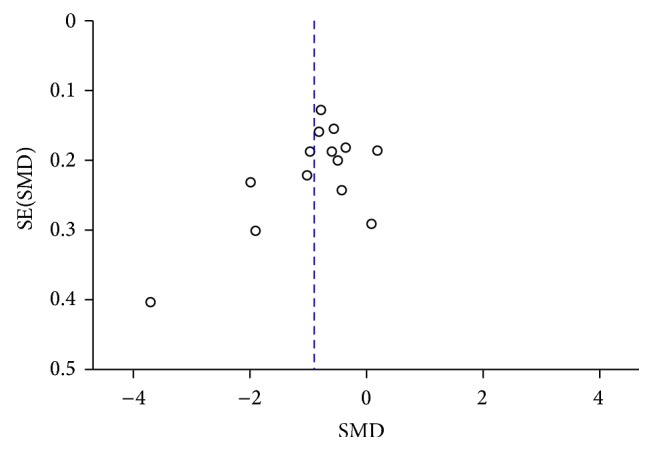
Funnel plot of CSF *α*-syn levels in PD compared to control.

**Figure 14 fig14:**
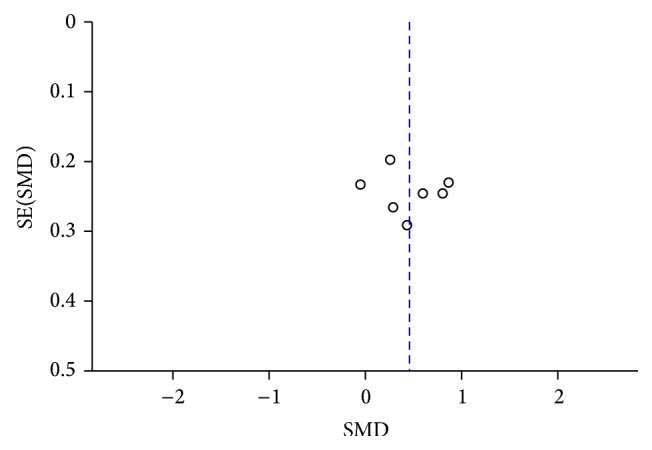
Funnel plot of CSF *α*-syn levels in PD compared to MSA.

**Figure 15 fig15:**
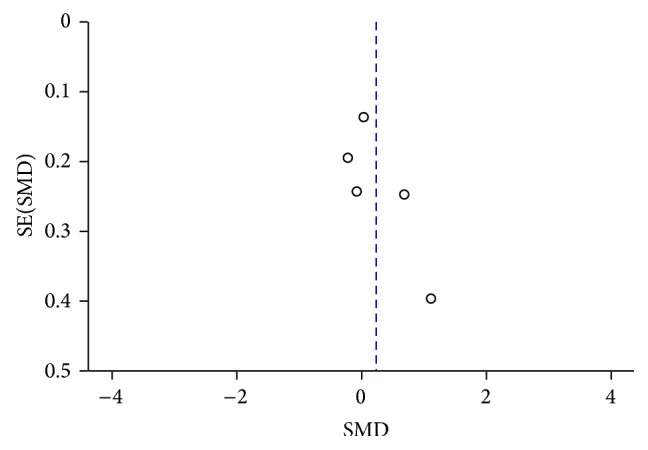
Funnel plot of CSF *α*-syn levels in PD compared to DLB.

**Figure 16 fig16:**
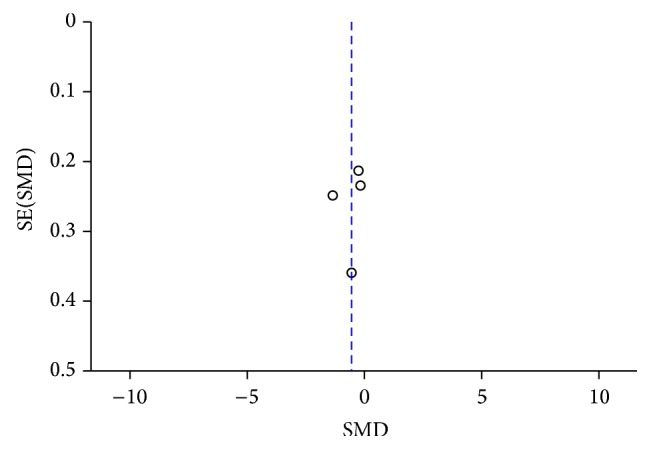
Funnel plot of CSF *α*-syn levels in PD compared to PSP.

**Figure 17:**
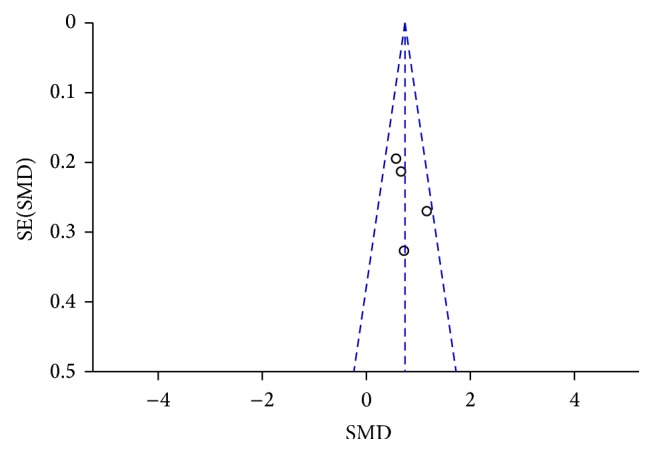
Funnel plot of CSF *α*-syn oligomers levels in PD compared to control.

A funnel plot was performed to assess publication bias of the literature, shown in Figures 13 [Fig fig14] [Fig fig15] [Fig fig16]–17. Visual inspection of every funnel plot indicated symmetrical distribution of SMD, suggesting no publication bias.

## 4. Discussion

### 4.1. The Function of *α*-Syn

*α*-syn is a neuronal protein of 140 amino acids and is normally localized in presynaptic terminals. It is the principal pathological hallmark of PD and other synucleinopathies such as MAS [7, 28]. Although the exact physiological function of *α*-syn remains to be defined, several studies have implicated its role in dopamine biosynthesis, synaptic plasticity, and vesicle dynamics. Evidence suggests that the overexpression of wild type or the mutants of *α*-syn might lead to a toxic gain of function related to alterations in axonal transport, oxidative stress, and neuroinflammation, which clearly suggest a primary involvement of this protein in neurodegeneration [29, 30]. These observations strongly suggest that *α*-syn aggregation is a critical factor in the etiology of genetic and sporadic PD and other types of Parkinsonism [31–33]. *α*-syn has been detected in biological fluids including CSF, plasma, and saliva [34, 35]. CSF reflects metabolic and pathological states of the central nervous system (CNS) more directly than any other body fluids, an optimal source of biomarkers for neurodegenerative diseases [22, 36]. The quantification of *α*-syn in CSF has been proposed as a potential biomarker for PD. Several studies have explored the potential use of *α*-syn as a PD diagnostic biomarker in human CSF, but the results are inconsistent with each other, thereby precluding judgment as to their clinical usefulness. Consequently, we conducted a meta-analysis in this paper.

### 4.2. The Diagnostic and Differential Diagnosis Efficacy of CSF *α*-Syn

Our results suggest the following. (1) CSF *α*-syn concentrations decreased in both PD and MSA, with the reduction in MSA being more significant, which might suggest that there is more widespread or faster neurodegeneration in MSA than in PD. The lower *α*-syn in CSF was not in accordance with neurons in PD and MSA. It may be explained by reduction in the release rate of *α*-syn into the extracellular space caused by intracellular aggregation [37]. (2) No difference in CSF *α*-syn concentration was identified between patients with PD and DLB. These results suggest that *α*-syn alone cannot be used to differentiate PD and DLB; it should be combined with other cognitive function related markers such as amyloid-*β* and tau. (3) Mean concentration of CSF *α*-syn was slightly decreased in PD compared to PSP, but the significance was marginal, and the sensitivity analysis indicated that the result was not stable and reliable; therefore more studies should be included for further analysis. (4) We further conducted a meta-analysis in CSF *α*-syn oligomeric levels in PD compared to controls (included non-UK Brain Bank diagnostic criteria, such as Hansson and Park's research); results indicated a trend toward higher CSF *α*-syn oligomeric concentrations in PD compared to control. This result was consistent with the pathological changes of oligomeric *α*-syn in neurons. Accordingly, oligomeric *α*-syn in CSF may represent a better biomarker for diagnosis in PD than *α*-syn [38, 39].

### 4.3. Reasons for the Inconsistent Result of Each Study

Many factors might contribute to the discordance of the results of CSF *α*-syn in PD: (1) heterogeneity of patients (including variability in patients' characteristics, such as age, disease duration, disease severity, and diagnosis criteria) and control groups (comprising healthy individuals and patients with cognitively normal who underwent lumbar puncture as a part of diagnostic work-up for other neurological conditions); (2) lack of widely used standardized operating procedures for CSF sample processing, including collection, handling, and storage; (3) inconsistent methods of detection and antibodies; (4) samples contaminated by blood. *α*-syn is highly abundant in blood, especially in erythrocytes, where levels are remarkably higher than plasma (and serum) and CSF [40]. If contamination by a low number of intact erythrocytes, which occurs in 10–20% of lumbar punctures, is not prevented in every CSF donor, it will falsely elevate CSF *α*-syn levels and thus skew study results [37].

### 4.4. The Advantages of This Study

Compared to the study by Zetterberg et al. [41, 42], the present study has the following advantages. (1) The latest literature was included. (2) In order to improve the reliability of the meta-analysis, strict limits of inclusion and exclusion criteria for the literature were defined. (3) This study used SMD for the meta-analysis instead of WMD (weighted mean difference), because *α*-syn concentration has a different metering method, such as means and medians. (4) This study not only compared PD with controls but also compared PD with the other Parkinsonian disorders, such as MSA, DLB, and PSP.

The present results showed that *α*-syn can be potentially used in diagnostic and differential diagnosis from MSA but not in differential diagnosis from DLB. Furthermore, oligomeric *α*-syn may represent a good biomarker for diagnosis in PD, but, combined with oligomeric *α*-syn, tau and amyloid-*β* would be better for differential synucleinopathies and tauopathies with Parkinsonism. In order to ensure more accurate and reliable analysis, future studies should link diagnostic criteria for PD (UK Brain Bank diagnostic criteria) and strictly limit the cerebrospinal fluid collection, storage, and testing standards.

## Figures and Tables

**Table 1:** Summary of studies included in the systematic review.

Author	Year	Detection method	PD		Control		MSA		DLB		PSA		References
			CSF		CSF		CSF		CSF		CSF		
			*N*	*α*-syn (ng/mL)	*N*	*α*-syn (ng/mL)	*N*	*α*-syn (ng/mL)	*N*	*α*-syn (ng/mL)	*N*	*α*-syn (ng/mL)	
Parnetti	2014	ELISA	44	22.15 (11.86–38.64)	25	36.5 (25.8–49.6)							[15]
Parnetti	2014	ELISA	71	22.00 (13.50–38.60)	45	39.67 (27.72–64.60)							[16]
Mondello	2014	ELISA	52	0.84 (0.69–1.01)	22	1.31 (0.94–1.5)	34	0.75 (0.64–1.06)			32	0.98 (0.79–1.2)	[17]
Van Dijk	2014	TR-FRET immunoassay	53	1.48 ± 0.41	50	1.70 ± 0.47							[18]
Mollenhauer	2013	ELISA	78	1.339 ± 0.236	48	1.504 ± 0.337							[19]
Aerts	2012	ELISA	58	26.0 (20.5–32.5)	57	25.0 (18.0–42.0)	47	25 (17–32)	24	24 (23–34)			[20]
Wang	2012	Luminex	83	0.389 ± 0.137	51	0.520 ± 0.130	14	0.330 ± 0.120			30	0.42 ± 0.186	[21]
			109	0.399 ± 0.137	71	0.487 ± 0.181	20	0.319 ± 0.114			22	0.419 ± 0.120	
Shi	2011	Luminex	126	0.38 ± 0.10	137	0.49 ± 0.17	20	0.30 ± 0.09					[22]
Parnetti	2011	ELISA	38	43.1 ± 47	32	68.9 ± 71			32	18.1 ± 16			[23]
Mollenhauer	2011	ELISA	273	1.34 ± 0.81	23	2.22 ± 1.31	15	1.11 ± 0.45	66	1.32 ± 0.62	8	1.78 ± 0.91	[24]
			51	1.19 ± 0.81	76	1.73 ± 1.83	29	1.24 ± 0.99	55	1.42 ± 1.26			
Hong	2010	Luminex	80	0.36 ± 0.10	91	0.47 ± 0.16							[25]
Öhrfelt	2009	ELISA	15	0.417 (0.246–0.522)	55	0.395 (0.298–0.452)			15	0.334 (0.220–0.406)			[26]

**Table 2:** Summary of studies included in the systematic review (*α*-syn oligomers).

Author	Year	Detection method	PD		Control		References
			*n*	*α*-syn oligomers	*n*	*α*-syn oligomers	
Park	2011	ELISA	23	0.33 ± 0.25	18	0.18 ± 0.11	[13]
Parnetti	2014	ELISA	71	4838.50 (2746.00–11196.50)	45	3284.50 (1664.50–11719.60)	[16]
Parnetti	2014	ELISA	44	4838 (3049–8141)	25	3139 (1500–6140)	[15]
Hansson	2014	Luminex	30	73,309 (36,361–326,297)	98	37,882 (21,763–136,685)	[27]
